# Comparative cardiovascular safety of GLP-1 receptor agonists versus other glucose-lowering agents in real-world patients with type 2 diabetes: a nationwide population-based cohort study

**DOI:** 10.1186/s12933-020-01053-0

**Published:** 2020-06-13

**Authors:** Chun-Ting Yang, Chen-Yi Yang, Huang-Tz Ou, Shihchen Kuo

**Affiliations:** 1 Institute of Clinical Pharmacy and Pharmaceutical Sciences, College of Medicine, National Cheng Kung University, 1, University Road, Tainan, 70101 Taiwan; 2 Department of Pharmacy, National Cheng Kung University Hospital, No. 138, Sheng Li Road, Tainan, 704 Taiwan; 3 Department of Pharmacy, College of Medicine, National Cheng Kung University, 1, University Road, Tainan, 70101 Taiwan; 4 Division of Metabolism, Endocrinology & Diabetes, Department of Internal Medicine, University of Michigan Medical School, 1500 E Medical Center Dr, Ann Arbor, MI 48109 USA

**Keywords:** GLP-1 receptor agonist, Cardiovascular safety, DPP-4 inhibitor, Sulfonylurea, Insulin

## Abstract

**Background:**

Current evidence about the cardiovascular safety of glucagon-like peptide-1 receptor agonist (GLP-1ra) possesses limited generalizability to real-world patients with type 2 diabetes (T2D) in usual practice. This study aimed to investigate the comparative cardiovascular safety of GLP-1ra in comparisons with dipeptidyl peptidase-4 inhibitor (DPP-4i), sulfonylurea (SU), and insulin in a real-world population with T2D.

**Methods:**

Adults with newly-diagnosed T2D were identified from Taiwan’s National Health Insurance Research Database in 2003–2014. A prevalent new-user cohort design was adopted to include a broad representation of real-world T2D patients being treated with GLP-1ra. The between-group comparability of baseline patient characteristics was achieved by matching on (1) initiation time of study drugs, (2) prior exposure to glucose-lowering agents, and (3) diabetes severity and complications, comorbidities, and concomitant cardiovascular medications using propensity scores. The primary outcome was a composite of cardiovascular disease (CVD) events and assessed up to the end of 2015. Cox modeling was employed to assess the association between study drugs and outcomes.

**Results:**

A total of 3195 GLP-1ra stable users was identified in 2011-2014. 1893, 1829, and 1367 GLP-1ra stable users were 1:1 matched to DPP-4i, SU and insulin users, respectively. Compared to DPP-4i, SU and insulin, the use of GLP-1ra was associated with a lower risk of composite CVD events [hazard ratio (95% confidence interval) 0.73 (0.57–0.96), 0.76 (0.57–1.00), and 0.81 (0.62–1.07), respectively]. Subgroup analyses revealed that GLP-1ra versus DPP-4i yielded a greater cardiovascular benefit in those without established CVD versus those with established CVD.

**Conclusions:**

This comparison study extends the supporting evidence for the cardiovascular safety of GLP-1ra to a broad spectrum of real-world T2D patients using GLP-1ra.

## Background

Although early initiation of glucagon-like peptide-1 receptor agonist (GLP-1ra) is recommended for patients with type 2 diabetes (T2D) who have established cardiovascular diseases (CVDs) or need to minimize hypoglycemia or promote weight loss [1], it is commonly observed that GLP-1ra is initiated in the later treatment course of T2D in real-world practice settings (especially after treatment failure under dual or triple glucose-lowering agents [GLAs] [2–6]). This may be due to several reasons. GLP-1ra is a newer class of GLAs, and thus patients with T2D have been exposed to various GLAs before GLP-1ra became available to them. Also, physicians’ or patients’ adoption of new drugs may not be as fast as expected due to uncertain long-term real-world drug effectiveness and safety evidence. For instance, a recent study found that less than 10% of the real-world patients who had comparable CVD risks with the participants in the Liraglutide Effect and Action in Diabetes: Evaluation of Cardiovascular Outcome Results (LEADER) trial were actually prescribed with liraglutide [7]. A great concern has been raised for the limited generalizability of the GLP-1ra cardiovascular outcomes trial (CVOT) results to the general T2D population [8]. Moreover, the injectable form of GLP-1ra likely discourages physicians or patients from the early initiation of GLP-1ra until treatment failure with multiple GLAs, although previous studies reported that patient adherence to GLP-1ra was conditioned by the number of daily drug doses [9, 10]. Also, considering the high acquisition cost of GLP-1ra, Taiwan’s National Health Insurance program implements a restricted reimbursement policy to limit the use of GLP-1ra only for those who have already failed to monotherapy with metformin or sulfonylurea, or to dual therapy with metformin and sulfonylurea.

CVOTs have found favorable cardiovascular effects associated with GLP-1ra [11–16], and the cardiovascular benefits of GLP-1ra and underlying mechanisms (e.g., reduction in left ventricular filling pressure or systemic inflammation, effect on endothelial function) have been documented [17–19] and summarized in the recent review and meta-analysis literature [20–22]. However, there are two important caveats about the findings of CVOTs: (1) study cohorts included in these randomized controlled trials (RCTs) were highly selective, thus reflecting only a subset of the real-world T2D population, and (2) the comparator drug in these RCTs was a placebo instead of an active GLA. In addition, although real-world evidence of the cardiovascular safety of GLP-1ra has emerged, the incident new-user cohort design was applied in existing studies to only include treatment-naïve users of GLP-1ra [23–28], which would limit the study generalizability and applicability. Furthermore, only a small portion or even none of Asian populations was included in published RCTs and observational studies. These highlight a research gap in the evaluation of the effects of GLP-1ra versus other classes of GLAs from the perspective of a more diverse T2D population in the real world, consisting of both patients who newly initiate GLP-1ra in their earlier T2D treatment courses and those who have failed with multiple GLAs and then switched to GLP-1ra.

The present study therefore evaluates the comparative cardiovascular effects of GLP-1ra in comparisons with dipeptidyl peptidase-4 inhibitor (DPP-4i), sulfonylurea (SU), and insulin using the prevalent new-user cohort design [29] to include a broad spectrum of real-world adults with T2D being treated with GLP-1ra to enhance the generalizability of study findings.

## Methods

### Data source

This was a retrospective cohort study utilizing Taiwan’s National Health Insurance Research Database (NHIRD) 2003–2015. The NHIRD is derived from the claims data of the National Health Insurance (NHI) program, which covers over 99% of Taiwan’s population (with approximately 23 million people insured) and provides de-identified longitudinal medical and prescription information for each enrolled beneficiary [30].

### Cohort identification

**Fig. 1:**
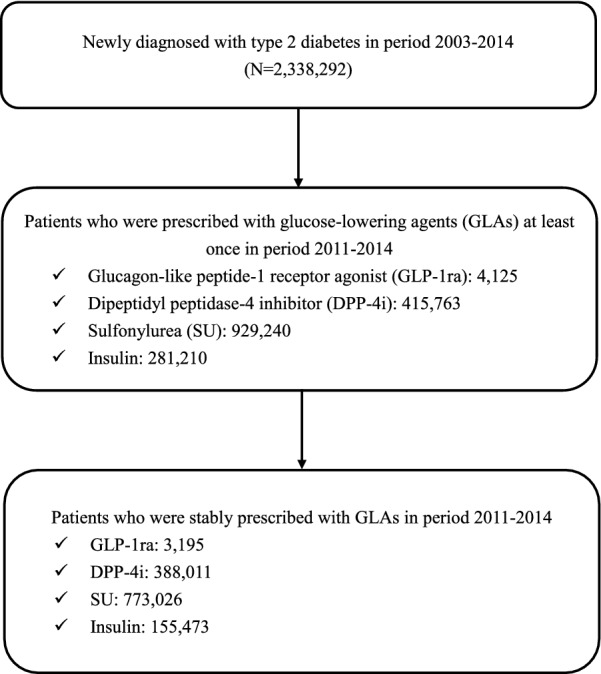
Flowchart of study population selection and identification

People with newly-diagnosed T2D were identified in 2003-2014 if they had: (1) at least one inpatient record with T2D diagnosis (International Classification of Diseases, Ninth Revision, Clinical Modification [ICD-9-CM]: 250.X0 or 250.X2, where X = 0–9), (2) at least two outpatient records with T2D diagnosis within a given year, or (3) one outpatient record with T2D diagnosis and prescription records of GLAs within a given year. Patients who were aged < 18 years at T2D diagnosis were excluded. Next, stable users of each study GLA (i.e., GLP-1ra, DPP-4i, SU, or insulin) in 2011–2014 were identified; the period 2011–2014 was chosen because GLP-1ra was reimbursed by the NHI program since 2011, and the period ended in 2014 to allow at least one-year follow-up for study subjects. Stable users were defined as patients who had at least one stable use set of the study GLA, which was defined as at least three sequential refills of the GLA after its initiation and a prescription gap between any two sequential refills was less than 30 days. A stable user of the study GLA can have multiple stable sets of that drug used chronologically. The study cohort selection flowchart is shown in Fig. 1.

To increase the chances of individual GLP-1ra users being matched with the most comparable users of comparators (i.e., DPP-4i, SU, or insulin) who had the most similar characteristics (i.e., T2D severity, complications, comorbidities, and prior treatments), we allowed stable use sets from a comparator GLA user to be re-used through a three-step matching process (Additional file 1: Figure S1). First, the index date was defined as the initiation date of GLP-1ra for GLP-1ra stable users or the first prescription date of stable use set for comparator GLA users in 2011-2014. Based on the availability of GLP-1ra in Taiwan (i.e., 2011), we aligned the cohort entry time for study groups to reduce immortal time bias and minimize confounding effects due to changes in the evolution of GLA treatment and clinical management over time. That is, for each GLP-1ra stable user, we identified stable use sets of a comparator GLA user with the index dates falling within ± 180 days surrounding the index date of the GLP-1ra user. Second, we adjusted previous utilization patterns/extent of all GLAs, including metformin, SU, meglitinides, thiazolidinediones, acarbose, DPP-4i, and insulin, at 1 year before GLP-1ra initiation. The utilization of individual GLAs was measured as the total day supply of a drug in the year before the index date. We matched previous utilization patterns/extent of GLAs between GLP-1ra and comparator GLA users, in which a maximum difference of 90 days supplied (i.e., ± 45 days) of each class of GLAs between groups was allowed. Lastly, the one-to-one seven-greedy propensity score matching (PSM) was used to adjust for the imbalance of baseline confounder patient characteristics between groups. The propensity score was estimated using a logistic regression model where treatment status (i.e., GLP-1ra versus comparator GLA) was treated as the dependent variable, and a comprehensive list of clinical characteristics (Table 1) related to either the selection of GLA treatment or study outcomes was identified as independent variables. The second and third steps above ensured that the between-group comparison derived from two groups of patients would have comparable disease severity and previous medication use patterns/extent. To minimize the computational demand for large sample size in the SU group (21,135,786 stable use sets of SU) in the matching process, we randomly sampled 30% of stable use sets of SU for matching with GLP-1ra stable users.

**Table 1:** Baseline patient characteristics for different glucose-lowering agent groups after the matching algorithm

Characteristics	GLP-1ra	1:1 matched DPP-4i	GLP-1ra	1:1 matched SU	GLP-1ra	1:1 matched insulin
Number of subjects	1893	1893	1829	1829	1367	1367
Age at index date (years, mean ± SD)	49.48 ± 11.61	51.78 ± 12.19	49.34 ± 11.64	51.27 ± 11.82	49.38 ± 12.06	53.07 ± 13.67^a^
Male at index date (%)	47.97	47.17	46.04	49.7	41.7	51.43
Diabetes duration^b^ (years, mean ± SD)	6.18 ± 2.74	6.46 ± 2.7	5.9 ± 2.82	6.32 ± 2.74	6.37 ± 2.75	6.33 ± 2.78
Comorbidity history (%)						
Hypertension	62.60	62.07	61.73	62.66	59.77	67.08
Hyperlipidemia	70.79	71.05	70.42	70.59	69.42	70.37
Stroke or transient ischemic attack	4.86	5.23	4.37	6.07	5.27	5.05
Heart failure	2.85	3.49	2.19	3.17	3.58	3.44
Myocardial infarction	1.53	1.06	1.20	1.53	1.32	2.19
Ischemic heart diseases	12.20	11.62	12.3	13.83	13.90	14.26
CIC category (%)						
Cancer	4.07	5.65	4.48	5.14	5.34	5.78
Gastrointestinal	27.63	26.62	25.48	26.46	26.63	30.43
Musculoskeletal	32.70	34.28	33.13	32.31	34.67	35.70
Pulmonary	7.82	8.45	7.27	8.37	7.39	11.49
Substance abuse complexity	2.54	3.06	2.41	2.62	3.00	2.49
Mental illness	8.51	8.82	8.75	10.5	10.02	10.02
Diabetes-related complications (%)						
Retinopathy	17.91	17.38	16.79	18.48	21.58	19.09
Nephropathy	27.21	27.63	24.93	27.23	27.58	28.75
Neuropathy	14.37	15.27	13.61	15.36	16.83	17.56
Peripheral vascular diseases	4.75	4.54	4.54	4.81	4.90	5.78
Cerebrovascular diseases	3.96	4.38	3.39	5.03	3.95	4.39
Cardiovascular diseases	14.95	14.69	14.71	17.00	16.46	18.07
Metabolic complications	0.85	2.17	1.04	1.48	2.56	2.71
Number of glucose-lowering agents prescribed one year before index date	3.19	3.34	3.22	3.56	3.21	3.39
Glucose-lowering agents one year before index date (MPR, mean ± SD)^c^						
Metformin	0.50 ± 0.43	0.50 ± 0.43	0.59 ± 0.41	0.59 ± 0.41	0.46 ± 0.43	0.46 ± 0.43
Sulfonylurea	0.43 ± 0.43	0.43 ± 0.43	0.66 ± 0.37	0.67 ± 0.37	0.32 ± 0.41	0.32 ± 0.41
Meglitinide	0.04 ± 0.18	0.04 ± 0.18	0.02 ± 0.10	0.02 ± 0.10	0.05 ± 0.19	0.05 ± 0.19
Thiazolidinedione	0.12 ± 0.28	0.12 ± 0.28	0.13 ± 0.30	0.14 ± 0.30	0.08 ± 0.23	0.08 ± 0.23
Acarbose	0.15 ± 0.31	0.15 ± 0.31	0.13 ± 0.29	0.13 ± 0.29	0.13 ± 0.28	0.13 ± 0.28
DPP-4i	0.67 ± 0.34	0.67 ± 0.35	0.42 ± 0.42	0.42 ± 0.42	0.34 ± 0.40	0.34 ± 0.40
Insulin	0.24 ± 0.38	0.24 ± 0.38	0.21 ± 0.36	0.21 ± 0.37	0.67 ± 0.36	0.67 ± 0.36
CVD-related medication history (%)						
Lipid-modifying agents	68.94	68.57	66.21	67.2	67.15	69.35
α-Blockers	4.12	4.28	3.50	2.90	3.80	4.10
β-Blockers	31.91	30.85	31.66	31.93	33.21	35.48
Agents acting on RAAS	43.69	42.37	45.05	44.18	42.28	43.75
Diuretics	18.28	18.01	20.01	20.07	21.36	18.36
Calcium channel blockers	32.86	31.01	32.75	33.84	32.92	35.92
Antiarrhythmics	1.37	1.85	1.31	1.91	1.32	2.12
Cardiac glycosides	0.69	1.43	0.77	0.82	0.80	1.54
Vasodilators used in cardiac diseases	8.19	9.67	7.93	9.51	9.73	10.31
Anti-platelets	28.84	30.27	28.05	31.00	30.94	33.50
Anti-coagulants	1.16	1.69	0.87	1.09	1.61	1.76

All confounders listed above were measured in the year prior to index date, except age, gender, and diabetes duration, which were determined at index date

*CIC* chronic illness with complexity, *CVD* cardiovascular disease, *DPP-4i* dipeptidyl peptidase-4 inhibitor, *GLP-1ra* glucagon-like peptide-1 receptor agonist, *MPR* medication possession ratio, *RAAS* renin–angiotensin–aldosterone system, *SD* standard deviation, *SU* sulfonylurea

^a^A significant difference between GLP-1ra and insulin users, as indicated by absolute standardized mean difference > 0.2

^b^Diabetes duration was measured as the time from the first date of type 2 diabetes diagnosis to index date

^c^MPR was measured as the sum of prescription refill days in the year prior to index date divided by 365

### Operational definitions of exposure and outcomes

The exposure to GLAs was measured using the World Health Organization Anatomical Therapeutic Chemical Classification system. The primary outcome was the composite CVDs with fatal/non-fatal events of myocardial infarction (MI), ischemic heart disease, heart failure, ischemic and hemorrhagic stroke, cardiogenic shock, sudden cardiac arrest, arteriosclerotic cardiovascular disease, or arrhythmia. Secondary outcomes included (1) all-cause death, (2) fatal CVDs, and (3) three-point major adverse cardiovascular event (MACE), including non-fatal MI, non-fatal stroke, or fatal CVDs. Using ICD-9-CM codes, cardiovascular outcomes were identified from inpatient and emergency department claims files in the NHIRD. The accuracy of ICD-9-CM coding for study outcomes in the NHIRD has been validated in previous studies [31–35]. The mortality status was ascertained from death cause records from the Ministry of Health and Welfare. Detailed information of the operational definitions is provided in Additional file 2: Table S1. Each patient was followed from the index date until the occurrence of study outcomes, discontinuation of study drugs, death, lost to follow-up from the NHI program, or the end of 2015, whichever came first.

### Statistical analysis

Baseline patient characteristics were measured at 1 year before or at the index date. Differences in baseline patient characteristics between study groups were compared using the standardized mean difference (SMD), where SMD values > 0.2 were considered to indicate a statistically significant difference between groups [36, 37]. Cox proportional hazard models were used to estimate the risk of study outcomes between GLP-1ra and comparator GLAs. Further, subgroup analyses were performed by including interaction terms of study groups and clinical characteristics as covariates in Cox models. A sensitivity analysis was performed where the cutoff point of statistically significant differences in baseline patient characteristics between the drug groups was re-defined as SMD > 0.1 and thus those variables remaining significantly different after PSM were further adjusted in Cox models. A two-tail *p*-value of less than 0.05 was considered statistically significant. All analyses were performed using SAS software version 9.4.

## Results

There were 3195 stable users of GLP-1ra identified in the period 2011-2014. Before the matching, the GLP-1ra users were generally younger, with a higher proportion being hyperlipidemic and a lower proportion having existing CVDs, compared to the comparator GLA users (Additional file 3: Table S2.). After the matching algorithm was applied, there were a total of 1893, 1829, and 1367 matched pairs of GLP-1ra users with DPP-4i, SU, and insulin users, respectively. There was no significant difference in baseline patient characteristics between the study groups after the matching, except that the GLP-1ra users were significantly younger than insulin users (Table 1). The average length of follow-up for study subjects ranged from 1.5 to 2 years.

Table 2 shows that the event rates of all four study outcomes in GLP-1ra users were lower than those in the three other matched GLA groups. For example, the event rates of the primary CVD composite outcome for the use of GLP-1ra versus the use of DPP-4i, SU, and insulin were 34.25 vs. 46.20, 31.10 vs. 40.24, and 43.31 vs. 65.88 per 1000 person-years, respectively.

**Table 2:** Event rates of study outcomes associated with the use of GLP-1ra versus other glucose-lowering agents

	GLP-1ra (n = 1893)	1:1 matched DPP-4i (n = 1893)	GLP-1ra (n = 1829)	1:1 matched SU (n = 1829)	GLP-1ra (n = 1367)	1:1 matched insulin (n = 1367)
Composite CVD^a^						
Number of events	92	141	83	127	78	171
Total person-years in follow-up	2686.18	3051.80	2669.19	3155.88	1800.99	2595.79
Crude rate (per 1000 person-years)	34.25	46.20	31.10	40.24	43.31	65.88
All-cause mortality						
Number of events	1	22	2	6	2	30
Total person-years in follow-up	2755.76	3188.36	2737.90	3275.08	1855.12	2793.32
Crude rate (per 1000 person-years)	0.36	6.90	0.73	1.83	1.08	10.74
Fatal CVD						
Number of events	1	13	2	3	1	9
Total person-years in follow-up	2755.76	3188.76	2737.90	3275.21	1855.16	2794.25
Crude rate (per 1000 person-years)	0.36	4.08	0.73	0.92	0.54	3.22
MACE^b^						
Number of events	28	59	29	43	21	68
Total person-years in follow-up	2740.75	3148.35	2721.35	3235.44	1843.70	2722.47
Crude rate (per 1000 person-years)	10.22	18.74	10.66	13.29	11.39	24.98

*CVD* cardiovascular disease, *DPP-4i* dipeptidyl peptidase-4 inhibitor, *GLP-1ra* glucagon-like peptide-1 receptor agonist, *MACE* major adverse cardiovascular event, *SU* sulfonylurea

^a^Composite CVD was a composite outcome that included acute myocardial infarction, ischemic heart disease, heart failure, stroke, cardiogenic shock, sudden cardiac arrest, arteriosclerotic cardiovascular disease, and arrhythmia

^b^Three-point MACE included non-fatal myocardial infarction, non-fatal stroke, and death due to cardiovascular diseases

**Fig. 2:**
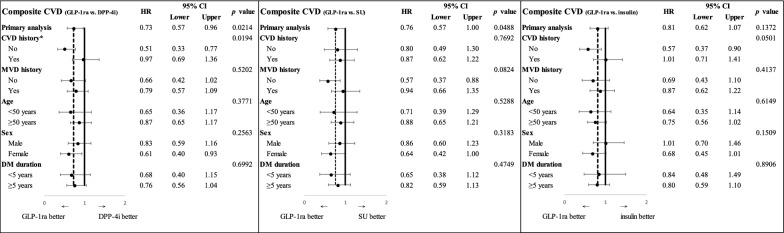
Primary and subgroup analyses for composite CVD associated with GLP-1ra versus other glucose-lowering agents^a^. CVD, cardiovascular disease; DM, diabetes mellitus; DPP-4i, dipeptidyl peptidase-4 inhibitor; GLP-1ra, glucagon-like peptide-1 receptor agonist; HR, hazard ratio; MVD, microvascular disease; SU, sulfonylurea. ^a^Composite CVD was a composite outcome that included acute myocardial infarction, ischemic heart disease, heart failure, stroke, cardiogenic shock, sudden cardiac arrest, arteriosclerotic cardiovascular disease, and arrhythmia. *In the testing of interaction in subgroup analyses, a *p* value of less than 0.05 was considered statistically significant

As illustrated in Fig. 2, primary analyses show that the hazard ratios (HRs) and 95% CIs for the composite CVD were 0.73 (0.57–0.96; *p *= .0214), 0.76 (0.57–1.00; *p *= .0488), 0.81 (0.62–1.07; *p *= .14) for GLP-1ra use versus DPP-4i, SU, and insulin use, respectively. As shown in Additional file 4: Figure S2, the assessment of three-point MACE reveals that the HRs and 95% CIs for GLP-1ra use versus DPP-4i, SU, and insulin use were 0.55 (0.35–0.86; *p *= .0087), 0.79 (0.49–1.26; *p *= .32), 0.62 (0.37–1.02; *p *= .06), respectively.

The results of subgroup analyses based on five patient characteristics (i.e., prior history of CVDs, prior history of microvascular diseases, age, gender, and diabetes duration) for the cardiovascular effect of GLP-1ra versus other GLAs are shown in Fig. 2 and Additional file 4: Figure S2 There was some heterogeneity for the primary outcome; a significant interaction was observed for the absence versus presence of established CVDs at baseline, with a greater benefit in reducing the composite CVD of using GLP-1ra versus DPP-4i for those without established CVDs at baseline. In contrast, there was a consistent benefit of GLP-1ra versus other GLAs on three-point MACE across all subgroups. In addition, as shown in original primary and subgroup analyses, sensitivity analyses using a different cutoff point for SMD (i.e., > 0.1) in PSM reveal consistent results that compared to three other GLAs, GLP-1ra was associated with a lower risk of the composite CVD and three-point MACE (Additional file 5: Table S3 and Additional file 6: Table S4).

## Discussion

This study included a broad representation of the real-world T2D population being treated with GLP-1ra by using the prevalent new-user cohort design to assess its cardiovascular safety compared to three commonly-used classes of GLAs in Taiwan. The majority (87%) of our study population was those who had failed with more than two types of GLAs and then initiated GLP-1ra. Our results show that GLP-1ra was associated with: (1) a significant cardiovascular benefit in the composite CVD outcome compared to DPP-4i and SU and a non-significantly lower risk of that compared to insulin, and (2) a significant lower risk of three-point MACE compared to DPP-4i and a non-significantly lower risk of that compared to SU and insulin. In addition, prior CVD history was a significant effect-modifier in the association between the use of GLP-1ra versus DPP-4i and the risk of the composite CVD outcome.

Currently, there are no published large, long-term RCTs to evaluate the comparative CVD safety of GLP-1ra in comparisons with other active GLAs. The Glycemia Reduction Approaches in Diabetes: A Comparative Effectiveness Study (GRADE) is an ongoing, pragmatic RCT to make head-to-head comparisons of GLP-1ra, DPP-4i, SU, and basal insulin in metformin-monotherapy patients with relatively recently-diagnosed T2D on glycemia-lowering effectiveness and patient-centered outcomes [38]. However, the GRADE may not be sufficiently powered to evaluate cardiovascular outcomes, and the GRADE participants are initiated with GLP-1ra in their earlier GLA treatment courses and are not representative of real-world patients.

### Real-world evidence on the comparative cardiovascular safety of GLP-1ra versus other GLAs

There is the emerging but limited real-world evidence on the comparative cardiovascular safety of GLP-1ra [23–28]. Among these studies, while two of them [23, 24] included only exenatide in the GLP-1ra user group in comparison with other GLA user groups, four other real-world observational studies are more relevant to our research. O’Brien et al. used the US nationwide administrative claims data for a retrospective cohort study among insured adults with T2D who newly started second-line GLAs after taking either metformin alone or no prior GLA, and found that the risk of composite CVD events (including ischemic heart disease, congestive heart failure, stroke, or peripheral artery disease, but not cardiovascular death) was significantly lower in GLP-1ra users compared to DPP-4i users (HR: 0.78, 95% CI 0.63–0.96) [25]. Svanström et al. performed a nationwide register-based cohort study in Denmark and Sweden, which included patients with T2D who newly initiated liraglutide or DPP-4i, and found that the risk of three-point MACE was significantly lower in liraglutide users compared to DPP-4i users (HR: 0.90, 95% CI 0.83–0.98) [26]. Mogensen et al. conducted a nationwide register-based cohort study in Denmark, which included T2D patients without prior MI or stroke that were newly initiated with a combination of metformin with GLP-1ra or SU, and revealed that the risk of three-point MACE was non-significantly lower in GLP-1ra users compared to SU users (HR: 0.82, 95% CI 0.55–1.21) [27]. Patorno et al. conducted a cohort study using a US commercial health plan database performed comparative analysis of propensity-score-matched incident new users of GLP-1ra versus DPP-4i, SU, and insulin, and reported non-significant differences in the composite CVD events (including acute myocardial infarction, unstable angina, ischemic or hemorrhagic stroke, or coronary revascularization, but not cardiovascular death) between study groups; the HRs (95% CI) were 1.20 (0.76–1.89), 1.05 (0.63–1.74), and 1.01 (0.73–1.41) for GLP-1ra versus DPP-4i, SU, and insulin, respectively [28].

Noticeably, these previous studies used the incident new-user cohort design to only include patients who newly initiated GLP-1ra or the comparator GLAs in the earlier treatment course of T2D, whereas our study utilized the prevalent new-user cohort design to additionally include patients who initiated GLP-1ra or the comparator GLAs in the later treatment course of T2D. For example, the majority of the previous study cohort [25, 27, 28] was only on metformin monotherapy before GLP-1ra initiation, whereas most of our study population had already received two or more GLAs before using GLP-1ra. Furthermore, the proportion of patients with comorbidities (e.g., dyslipidemia) and macro- and micro-vascular complications (e.g., CVDs, nephropathy, neuropathy, and retinopathy) in the previous studies [26, 28] was lower than that in our study population. Thus, adding to previous studies that focused on the cardiovascular effects of GLP-1ra in the earlier treatment course of T2D, we extend evidence on the cardiovascular safety profile of GLP-1ra to support its rational use in a broader spectrum of real-world T2D patients being treated with GLP-1ra.

### Interaction between CVD history and GLP-1ra versus DPP-4i use on study outcomes

Moreover, we observed a significant interaction between the status of prior CVD history and the use of GLP-1ra versus DPP-4i. Compared with DPP-4i, GLP-1ra significantly lowered the risk of composite CVD events by 49% in patients without established CVDs, but it had a non-significant risk reduction of 3% in those with established CVDs.

The interaction effect between the baseline CVD risk levels and GLP-1ra use on cardiovascular outcomes was also observed in the LEADER trial which showed a greater cardiovascular benefit associated with liraglutide among patients aged ≥ 50 years and with established CVD compared to those aged ≥ 60 years and with only risk factors for CVD [11]. However, in the REWIND trial [15], no interaction effect was observed between CVD history and the use of dulaglutide on three-point MACE.

The inconsistent results of subgroup analyses in our study, the LEADER and REWIND trials may be explained by the following reasons. First, the baseline CVD risk levels differed across study populations. The proportions of study patients with established CVD were about 20%, 31%, and 81% in the present study, the REWIND trial, and the LEADER trial, respectively. Second, the comparison group was different across these studies; the comparator of this study was three classes of active GLAs (i.e., DPP-4i, SU, and insulin), while the comparator in the LEADER and REWIND trials was placebo. Third, different classifications of study subgroups may also contribute to the discrepancies of study results. The LEADER trial classified the study population into the subgroup patients aged ≥ 50 years with established CVD versus those aged ≥ 60 years with only risk factors for CVD; however, the REWIND trial performed the subgroup analysis based on those with prior CVD history versus those without CVD history, which was similar to our study.

Although subgroup analysis results about cardiovascular effects of GLP-1ra varied by baseline CVD risk levels, the main conclusion of cardiovascular benefits associated with GLP-1ra use was consistently made across these studies. Future research is warranted to explore the heterogeneous treatment effects of GLP-1ra associated with baseline CVD risk levels on cardiovascular outcomes.

### Study strengths and limitations

The present study has several strengths. First, the implementation of the prevalent new-user cohort design to construct the study cohort is essential to ensure the comprehensiveness assessment of the role of GLP-1ra in a diverse real-world population of patients in the earlier or later treatment courses of T2D. It also ensures the completeness of the estimation of the real-world cardiovascular effects of GLP-1ra and greatly enhances the generalizability of this study to real-world settings. Second, a rigorous three-step matching algorithm was applied in our study cohort selection process to ensure comparability between study groups and to minimize potential time-related problems that are challenging in real-world comparative drug effect studies. Specifically, the index year matching, which aligns cohort entry time between study groups, enhanced comparability between study groups and reduced time-related bias (e.g., different clinical practices or advances in health technology over time). A matching procedure that achieves a balance in prior GLA history and disease conditions between study groups allows between-group comparisons starting from comparable status of diabetes severity, in which time-lag bias can be reduced [39, 40]. Third, prior GLA history in this study was measured based on the medication refill data, which could be a surrogate indicator for patients’ health behaviors (e.g., drug refill behaviors). For instance, patients who have high persistence or adherence to medication refills may be more likely to engage in healthier behaviors compared to those that do not [41]. Thus, adjustment for the medication refill pattern might allow us to control for potential variations in patients’ health behaviors between study groups and thus minimize potential healthy user bias and its effect on study estimates. Lastly, a series of subgroup and sensitivity analyses that considered plausible real-world scenarios of GLP-1ra use ensured the robustness of the study results.

Several limitations should also be acknowledged. First, like other observational studies using administrative claims data, the residual bias attributable to unmeasured confounders (e.g., physicians’ preferences, laboratory data) might exist. However, with careful adjustments of disease severity/conditions and prior GLA use, we may reduce the unmeasured confounding that is commonly seen in claims-based studies. Second, medication non-adherence (e.g., short-term or accidental use of drugs of interest) is challenging in real-world observational studies. We applied the stable user definition from our previous studies [42–47] to restrict the analysis to stable GLA users, which might eliminate potential bias introduced from the accidental use or non-adherence of a drug. Third, the generalizability of the study results may be limited to countries with universal health insurance coverage. Lastly, sodium-glucose co-transporter-2 inhibitors were not available in Taiwan’s National formulary before May 1, 2016, and thus were not included in analyses.

## Conclusions

Our study findings extend supporting evidence for the cardiovascular safety of GLP-1ra in comparisons with DPP-4i, SU, and insulin to a broad representation of the real-world T2D population using GLP-1ra. The use of GLP-1ra versus DPP-4i may yield a greater cardiovascular benefit in patients without established CVDs compared to those with established CVDs.

## Supplementary information

**Additional file 1.** Illustration of three-step matching algorithm.

**Additional file 2.** The WHO Anatomical Therapeutic Chemical (ATC) codes for classification of study drugs and ICD-9- CM codes for definition of study outcomes.

**Additional file 3.** Baseline patient characteristics for different glucose-lowering agent groups before the matching algorithm.

**Additional file 4.** Primary and subgroup analyses for three-point major adverse cardiovascular event associated with the use of GLP-1ra versus other glucose-lowering agents.

**Additional file 5.** Primary and subgroup analyses of hazard ratios (95% CI) for composite CVD associated with the use of GLP-1ra versus other glucose-lowering agents.

**Additional file 6.** Primary and subgroup analyses of hazard ratios (95% CI) for three-point major adverse cardiovascular event associated with the use of GLP-1ra versus other glucose-lowering agents.

## Data Availability

Data sharing is not applicable to this study as data management and analysis were only allowed to be conducted in Health and Welfare Data Science Center in Taiwan for data privacy and safety concerns.

## Abbreviations

CVD: Cardiovascular disease
CVOT: Cardiovascular outcomes trial
DPP-4i: Dipeptidyl peptidase-4 inhibitor
GLA: Glucose-lowering agent
GLP-1ra: Glucagon-like peptide-1 receptor agonist
GRADE: Glycemia reduction approaches in diabetes: a comparative effectiveness study
ICD-9-CM: International classification of diseases, ninth revision, clinical modification
MACE: Major adverse cardiovascular event
NHI: National Health Insurance
NHIRD: National Health Insurance Research Database
RCT: Randomized controlled trial
SMD: Standardized mean difference
SU: Sulfonylurea
T2D: Type 2 diabetes
