# Supplementary material for: Comparative cardiovascular safety of GLP-1 receptor agonists versus other glucose-lowering agents in real-world patients with type 2 diabetes: a nationwide population-based cohort study
Source: Cardiovasc Diabetol. 2020 Jun 13;19:83. doi: 10.1186/s12933-020-01053-0 (PMC7293792; doi:10.1186/s12933-020-01053-0)
Supplement: Supplementary file 1 — Additional file 1. Illustration of three-step matching algorithm. [file 12933_2020_1053_MOESM1_ESM.docx]

Figure S1: Illustration of three-step matching algorithm


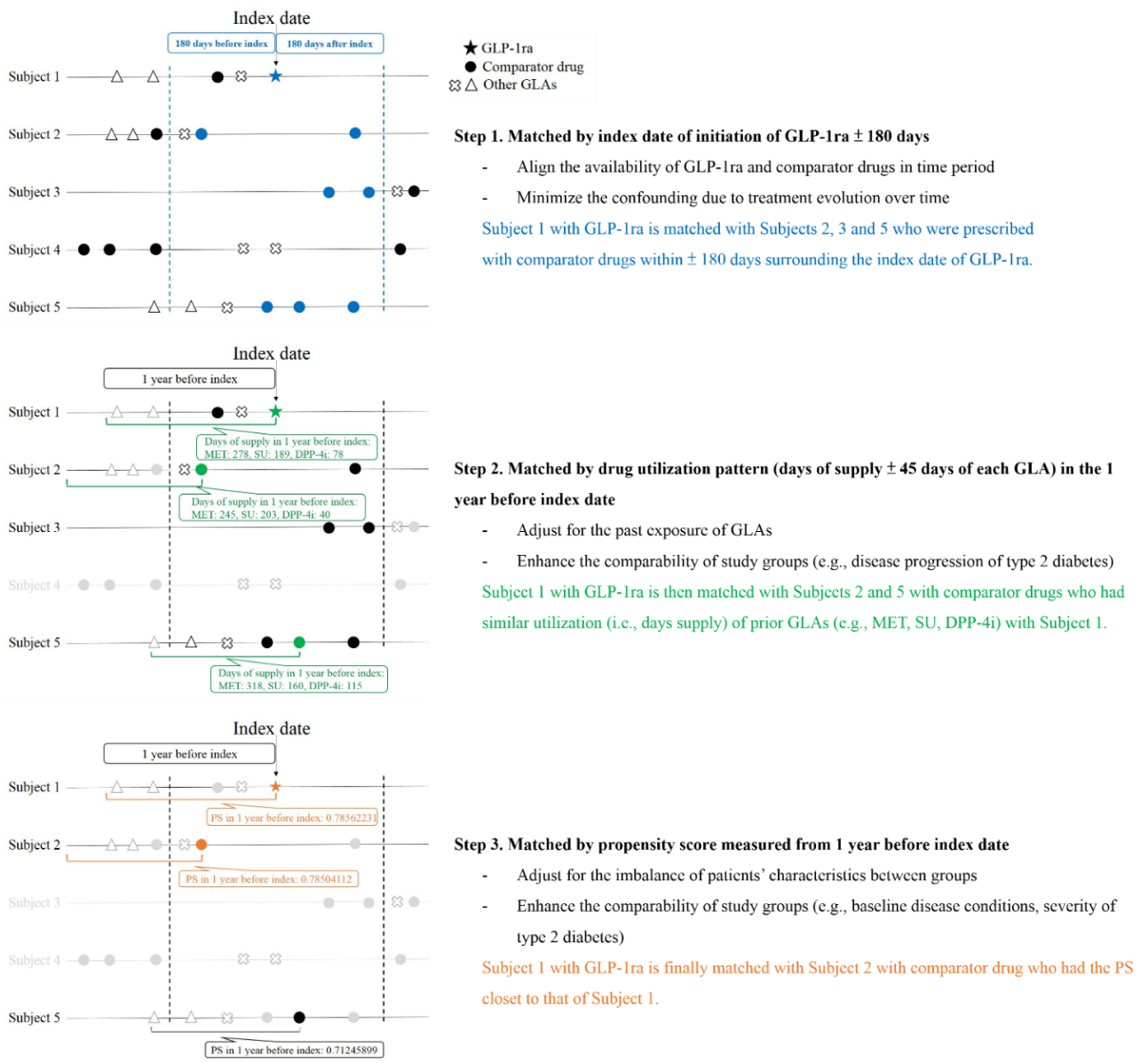


Abbreviations: GLP-1ra, glucagon-like peptide-1 receptor agonist; GLA, glucose-lowering agent; MET, metformin; SU, sulfonylurea; DPP-4i, dipeptidyl peptidase-4 inhibitor; PS, propensity score.
