# Supplementary material for: Comparative cardiovascular safety of GLP-1 receptor agonists versus other glucose-lowering agents in real-world patients with type 2 diabetes: a nationwide population-based cohort study
Source: Cardiovasc Diabetol. 2020 Jun 13;19:83. doi: 10.1186/s12933-020-01053-0 (PMC7293792; doi:10.1186/s12933-020-01053-0)
Supplement: Supplementary file 2 — Additional file 2. The WHO Anatomical Therapeutic Chemical (ATC) codes for classification of study drugs and ICD-9- CM codes for definition of study outcomes. [file 12933_2020_1053_MOESM2_ESM.docx]

Table S1: The WHO Anatomical Therapeutic Chemical (ATC) codes for classification of study drugs and ICD-9-CM codes for definition of study outcomes

| Study exposure and outcomes | Operational definitions | |
| --- | --- | --- |
| Classification of glucose-lowering agents | **Anatomical Therapeutic Chemical (ATC) codes** | |
| Metformin | A10BA02 |  |
| Sulfonylurea | First 5 characters: A10BB |  |
| Meglitinide | A10BX02, A10BX03 |  |
| Acarbose | A10BF01 |  |
| Thiazolidinedione | First 5 characters: A10BG |  |
| Dipeptidyl peptidase-4 inhibitor | First 5 characters: A10BH |  |
| Glucagon-like peptide-1 receptor agonist | First 5 characters: A10BJ |  |
| Cardiovascular outcomes | **ICD-9-CM disease codes** | **ICD-9-CM procedure codes** |
| Acute myocardial infarction | 410 |  |
| Ischemic heart disease | 411, 413, 414, V45.81, V45.82 | 36.0, 36.1, 36.2, 36.3, 36.9, 88.5, 00.66 |
| Heart failure | 428 |  |
| Stroke or transient ischemic attack | 430-437, V12.54 | 00.61, 00.63, 38.11, 38.12 |
| Cardiogenic shock | 785.51 |  |
| Sudden cardiac arrest | V12.53 |  |
| Arteriosclerotic cardiovascular disease | 429.2 |  |
| Arrhythmia | 426, 427 |  |
