# Supplementary material for: Comparative cardiovascular safety of GLP-1 receptor agonists versus other glucose-lowering agents in real-world patients with type 2 diabetes: a nationwide population-based cohort study
Source: Cardiovasc Diabetol. 2020 Jun 13;19:83. doi: 10.1186/s12933-020-01053-0 (PMC7293792; doi:10.1186/s12933-020-01053-0)
Supplement: Supplementary file 3 — Additional file 3. Baseline patient characteristics for different glucose-lowering agent groups before the matching algorithm. [file 12933_2020_1053_MOESM3_ESM.docx]

Table S2: Baseline patient characteristics for different glucose-lowering agent groups before the matching algorithm^a^

| **Characteristics** | **GLP-1ra** | **DPP-4i** | **SU** | **Insulin** |
| --- | --- | --- | --- | --- |
| Number of subjects | 3,195 | 7,665,432 | 6,340,894 | 3,799,662 |
| Age at index date (years, mean ± SD) | 48.61 ± 11.92 | 62.44 ± 12.35^b^ | 62.39 ± 11.96^b^ | 58.15 ± 15.87^b^ |
| Male at index date (%) | 45.57 | 53.76 | 53.19 | 53.04 |
| Diabetes duration^c^ (years, mean ± SD) | 5.99 ± 2.81 | 6.07 ± 2.81 | 5.47 ± 2.77 | 6.41 ± 2.77 |
| Comorbidity history (%) |  |  |  |  |
| Hypertension | 61.16 | 67.37 | 66.98 | 61.37 |
| Hyperlipidemia | 70.14 | 58.37^b^ | 57.10^b^ | 53.49^b^ |
| Stroke or transient ischemic attack | 4.48 | 11.68^b^ | 9.39 | 12.51^b^ |
| Heart failure | 2.69 | 5.39 | 3.28 | 6.85 |
| Myocardial infarction | 1.28 | 2.38 | 1.34 | 2.25 |
| Ischemic heart diseases | 11.99 | 19.71^b^ | 15.33 | 17.76 |
| CIC category (%) |  |  |  |  |
| Cancer | 4.41 | 7.74 | 6.88 | 8.21 |
| Gastrointestinal | 26.23 | 26.60 | 27.08 | 28.37 |
| Musculoskeletal | 33.83 | 38.69 | 40.53 | 34.50 |
| Pulmonary | 7.86 | 9.46 | 9.31 | 10.87 |
| Substance abuse complexity | 2.47 | 1.66 | 1.64 | 3.36 |
| Mental illness | 9.45 | 9.97 | 10.25 | 11.13 |
| Diabetes-related diseases (%) |  |  |  |  |
| Retinopathy | 17.15 | 15.00 | 11.26 | 24.15 |
| Nephropathy | 26.04 | 26.11 | 17.57^b^ | 37.82^b^ |
| Neuropathy | 13.83 | 13.75 | 11.61 | 19.95 |
| Peripheral vascular diseases | 4.54 | 5.22 | 4.77 | 8.06 |
| Cerebrovascular diseases | 3.60 | 9.85^b^ | 7.82 | 10.62^b^ |
| Cardiovascular diseases | 14.55 | 24.44^b^ | 18.86 | 22.86^b^ |
| Metabolic complications | 1.44 | 1.17 | 0.88 | 6.65^b^ |
| Number of glucose-lowering agents prescribes one year before index date | 2.94 | 2.78 | 2.34 | 2.50 |
| Glucose-lowering agents one year before index date (MPR, mean ± SD)^d^ |  |  |  |  |
| Metformin | 0.48 ± 0.43 | 0.47 ± 0.45 | 0.60 ± 0.43 | 0.31 ± 0.42 |
| Sulfonylurea | 0.40 ± 0.43 | 0.42 ± 0.44 | 0.82 ± 0.27 | 0.22 ± 0.37 |
| Meglitinide | 0.05 ± 0.19 | 0.07 ± 0.23 | 0.01 ± 0.08 | 0.06 ± 0.22 |
| Thizaolidinedione | 0.14 ± 0.30 | 0.09 ± 0.25 | 0.09 ± 0.26 | 0.06 ± 0.20 |
| Acarbose | 0.15 ± 0.30 | 0.14 ± 0.32 | 0.10 ± 0.27 | 0.11 ± 0.27 |
| Dipeptidyl peptidase-4 inhibitor | 0.41 ± 0.42 | 0.71 ± 0.33 | 0.13 ± 0.30 | 0.17 ± 0.33 |
| Insulin | 0.30 ± 0.41 | 0.07 ± 0.23 | 0.03 ± 0.15 | 0.78 ± 0.31 |
| CVD-related medication history (%) |  |  |  |  |
| Lipid modifying agents | 67.01 | 61.57 | 54.36^b^ | 55.11^b^ |
| α-blockers | 3.57 | 6.30 | 5.20 | 6.81 |
| β-blockers | 31.92 | 35.73 | 30.38 | 33.01 |
| Agents acting on RAAS (renin-angiotensin-aldosterone system) | 42.25 | 45.14 | 41.66 | 41.35 |
| Diuretics | 18.65 | 20.19 | 17.13 | 25.76 |
| Calcium channel blockers | 31.36 | 40.86 | 43.4^b^ | 39.21 |
| Antiarrhythmics | 1.22 | 2.59 | 1.86 | 2.63 |
| Cardiac glycosides | 0.75 | 2.22 | 1.64 | 2.53 |
| Vasodilators used in cardiac diseases | 8.26 | 14.44 | 10.20 | 15.17^b^ |
| Anti-platelets | 28.17 | 41.50^b^ | 34.67 | 39.52^b^ |
| Anti-coagulants | 1.06 | 1.93 | 1.23 | 1.87 |

Abbreviations: GLP-1ra, glucagon-like peptide-1 receptor agonist; DPP-4i, dipeptidyl peptidase-4 inhibitor; SU, sulfonylurea; SD, standard deviation; CIC, chronic illness with complexity; MPR, medication possession ratio; CVD, cardiovascular disease; RAAS, renin-angiotensin-aldosterone system.

^a^All confounders listed above were measured in the year prior to index date, except age, gender, and diabetes duration, which were determined at index date.

^b^A significant difference between GLP-1ra and insulin users, as indicated by absolute standardized mean difference > 0.2.

^c^Diabetes duration was measured as the time from the first date of type 2 diabetes diagnosis to index date.

^d^MPR was measured as the sum of prescription refill days in the year prior to index date divided by 365.
