# Supplementary material for: Comparative cardiovascular safety of GLP-1 receptor agonists versus other glucose-lowering agents in real-world patients with type 2 diabetes: a nationwide population-based cohort study
Source: Cardiovasc Diabetol. 2020 Jun 13;19:83. doi: 10.1186/s12933-020-01053-0 (PMC7293792; doi:10.1186/s12933-020-01053-0)
Supplement: Supplementary file 4 — Additional file 4. Primary and subgroup analyses for three-point major adverse cardiovascular event associated with the use of GLP-1ra versus other glucose-lowering agents. [file 12933_2020_1053_MOESM4_ESM.docx]

Figure S2: Primary and subgroup analyses for three-point major adverse cardiovascular event associated with the use of GLP-1ra versus other glucose-lowering agents


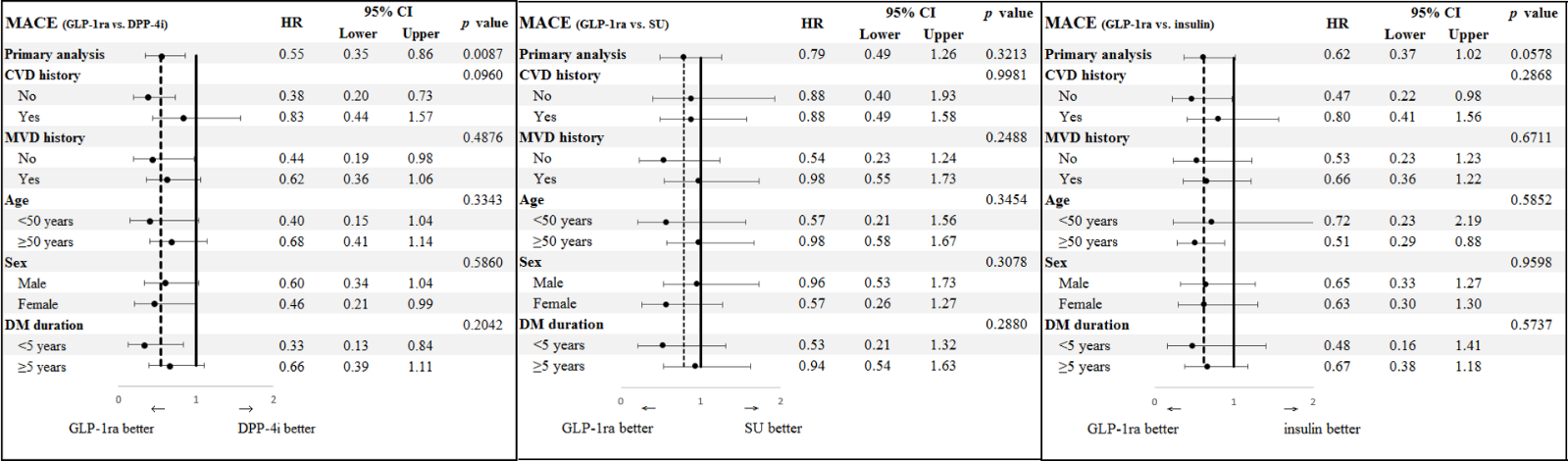


Abbreviations: MACE, major adverse cardiovascular event; HR, hazard ratio; GLP-1ra, glucagon-like peptide-1 receptor agonist; DPP-4i, dipeptidyl peptidase-4 inhibitor; SU, sulfonylurea; CVD, cardiovascular disease; MVD, microvascular disease; DM, diabetes mellitus.

Note: Three-point MACE included non-fatal myocardial infarction, non-fatal stroke, and death due to cardiovascular disease.
