# Supplementary material for: Comparative cardiovascular safety of GLP-1 receptor agonists versus other glucose-lowering agents in real-world patients with type 2 diabetes: a nationwide population-based cohort study
Source: Cardiovasc Diabetol. 2020 Jun 13;19:83. doi: 10.1186/s12933-020-01053-0 (PMC7293792; doi:10.1186/s12933-020-01053-0)
Supplement: Supplementary file 5 — Additional file 5. Primary and subgroup analyses of hazard ratios (95% CI) for composite CVD associated with the use of GLP-1ra versus other glucose-lowering agents. [file 12933_2020_1053_MOESM5_ESM.docx]

Table S3: Primary and subgroup analyses of hazard ratios (95% CI) for composite CVD associated with the use of GLP-1ra versus other glucose-lowering agents^a^

| **Composite CVD^b^** | **GLP-1ra vs. 1:1**  **matched DPP-4i** | **GLP-1ra vs. 1:1 matched SU** | **GLP-1ra vs.1:1 matched insulin** |
| --- | --- | --- | --- |
| Primary analysis | 0.89 (0.68, 1.16) | 0.86 (0.65, 1.13) | 0.84 (0.64, 1.11) |
| CVD history |  |  |  |
| No | 0.57 (0.37, 0.87)^c^ | 0.84 (0.52, 1.37) | 0.61 (0.39, 0.95) |
| Yes | 1.16 (0.82, 1.64)^c^ | 0.97 (0.69, 1.36) | 1.00 (0.71, 1.41) |
| MVD history |  |  |  |
| No | 0.80 (0.51, 1.24) | 0.67 (0.43, 1.05) | 0.72 (0.45, 1.15) |
| Yes | 0.91 (0.66, 1.27) | 1.00 (0.70, 1.44) | 0.90 (0.64, 1.25) |
| Age |  |  |  |
| <50 years | 0.65 (0.36, 1.17) | 0.71 (0.39, 1.29) | 0.65 (0.36, 1.17) |
| ≥50 years | 0.88 (0.65, 1.18) | 0.88 (0.65, 1.21) | 0.80 (0.59, 1.08) |
| Sex |  |  |  |
| Male | 0.93 (0.66, 1.31) | 0.97 (0.68, 1.4) | 1.02 (0.70, 1.47) |
| Female | 0.78 (0.51, 1.20) | 0.73 (0.47, 1.13) | 0.69 (0.46, 1.03) |
| DM duration |  |  |  |
| <5 years | 0.81 (0.48, 1.37) | 0.75 (0.44, 1.29) | 0.87 (0.49, 1.54) |
| ≥5 years | 0.91 (0.67, 1.24) | 0.90 (0.65, 1.25) | 0.84 (0.61, 1.14) |

Abbreviations: GLP-1ra, glucagon-like peptide-1 receptor agonist; DPP-4i, dipeptidyl peptidase-4 inhibitor; SU, sulfonylurea; CVD, cardiovascular disease; MVD, microvascular disease; DM, diabetes mellitus.

^a^All analyses above were adjusted for imbalanced patient characteristics between GLP-1ra and other glucose-lowering agent subgroups, as indicated by values of absolute standardized mean difference > 0.1.

^b^Composite CVD was a composite outcome that included acute myocardial infarction, ischemic heart disease, heart failure, stroke, cardiogenic shock, sudden cardiac arrest, arteriosclerotic cardiovascular disease, and arrhythmia.

^c^In the testing of interaction in subgroup analyses, a *p* value of less than 0.05 was considered statistically significant.
