# Supplementary material for: Comparative cardiovascular safety of GLP-1 receptor agonists versus other glucose-lowering agents in real-world patients with type 2 diabetes: a nationwide population-based cohort study
Source: Cardiovasc Diabetol. 2020 Jun 13;19:83. doi: 10.1186/s12933-020-01053-0 (PMC7293792; doi:10.1186/s12933-020-01053-0)
Supplement: Supplementary file 6 — Additional file 6. Primary and subgroup analyses of hazard ratios (95% CI) for three-point major adverse cardiovascular event associated with the use of GLP-1ra versus other glucose-lowering agents. [file 12933_2020_1053_MOESM6_ESM.docx]

Table S4: Primary and subgroup analyses of hazard ratios (95% CI) for three-point major adverse cardiovascular event associated with the use of GLP-1ra versus other glucose-lowering agents^a^

| **MACE^b^** | **GLP-1ra vs. 1:1 matched DPP-4i** | **GLP-1ra vs. 1:1 matched SU** | **GLP-1ra vs. 1:1 matched insulin** |
| --- | --- | --- | --- |
| Primary analysis | 0.65 (0.41, 1.03) | 0.90 (0.56, 1.44) | 0.62 (0.38, 1.03) |
| CVD history |  |  |  |
| No | 0.44 (0.23, 0.84) | 0.93 (0.42, 2.04) | 0.48 (0.23, 1.00) |
| Yes | 0.98 (0.51, 1.87) | 0.98 (0.54, 1.77) | 0.78 (0.40, 1.53) |
| MVD history |  |  |  |
| No | 0.52 (0.23, 1.19) | 0.63 (0.27, 1.47) | 0.53 (0.23, 1.24) |
| Yes | 0.70 (0.41, 1.21) | 1.03 (0.58, 1.84) | 0.67 (0.36, 1.23) |
| Age |  |  |  |
| <50 years | 0.40 (0.15, 1.05) | 0.57 (0.21, 1.57) | 0.76 (0.25, 2.32) |
| ≥50 years | 0.68 (0.41, 1.14) | 0.98 (0.58, 1.68) | 0.51 (0.29, 0.89) |
| Sex |  |  |  |
| Male | 0.66 (0.38, 1.16) | 1.09 (0.60, 1.97) | 0.62 (0.32, 1.22) |
| Female | 0.59 (0.27, 1.28) | 0.65 (0.29, 1.46) | 0.62 (0.30, 1.29) |
| DM duration |  |  |  |
| <5 years | 0.40 (0.16, 1.00) | 0.61 (0.24, 1.54) | 0.47 (0.16, 1.40) |
| ≥5 years | 0.78 (0.46, 1.31) | 1.03 (0.60, 1.80) | 0.68 (0.39, 1.20) |

Abbreviations: MACE, major adverse cardiovascular event; GLP-1ra, glucagon-like peptide-1 receptor agonist; DPP-4i, dipeptidyl peptidase-4 inhibitor; SU, sulfonylurea; CVD, cardiovascular disease; MVD, microvascular disease; DM, diabetes mellitus.

^a^All analyses above were adjusted for imbalanced patient characteristics between GLP-1ra and other glucose-lowering agent subgroups, as indicated by values of absolute standardized mean difference > 0.1.

^b^Three-point MACE included non-fatal myocardial infarction, non-fatal stroke, and death due to cardiovascular disease.
